# Knowledge, attitude and practices related to the use of personal protective equipment among welders in small-scale metal workshops in Nansana Municipality, Wakiso District, Uganda

**DOI:** 10.1080/21642850.2022.2106987

**Published:** 2022-08-05

**Authors:** Aisha Nalugya, Juliet Kiguli, Solomon T. Wafula, Rebecca Nuwematsiko, Richard K. Mugambe, Patience Oputan, Arnold Tigaiza, John Bosco Isunju, Tonny Ssekamatte

**Affiliations:** a Department of Disease Control and Environmental Health, Makerere University School of Public Health, Kampala, Uganda; b Department of Community Health and Behavioural Sciences, Makerere University School of Public Health, Kampala, Uganda

**Keywords:** Welders, occupational health, safety, hazards, PPE

## Abstract

**Background:**

Despite the risks involved in welding, there is limited evidence of the knowledge, attitude and practices related to the use of personal protective equipment (PPE) among welders in small-scale metal workshops. We assessed the level of knowledge, attitude and practices (KAP) related to PPE use among welders in small-scale workshops in Nansana Municipality, Wakiso district, Uganda.

**Methods:**

A cross-sectional study was conducted among 329 welders. A structured questionnaire was used to collect data on welder knowledge and attitude while an observation checklist was used to establish utilization of PPE. Ten key informant interviews were conducted to further understand welders’ knowledge, attitude and PPE-related practices. A ‘modified Poisson’ regression analysis was done to establish the independent factors associated with PPE KAP. NVivo 12 was used for the management of qualitative data. A thematic content approach guided qualitative data analysis.

**Results:**

About 61.4% (202/329) of the respondents had a high level of knowledge, 68.7% (226/329) had a negative attitude, and only 37.1% (122/329) exhibited good PPE-related practices. Knowledge of PPE use was associated with the type of training (aPR = 1.52, CI = 1.29–1.80). Attitude toward PPE use was associated with the level of education (aPR = 2.31, CI = 1.28–4.14), duration of work experience (aPR = 2.37, CI = 1.01–5.55), (aPR = 2.79, CI = 1.13–6.89), and level of knowledge (aPR = 1.73, CI = 1.13–2.65). PPE-related practices were associated with the type of training (aPR = 2.91, CI = 2.14–3.96) and attitude (aPR = 1.45, CI = 1.08–1.93).

**Conclusion:**

While the welders’ level of knowledge of PPE was found to be high, the majority exhibited a negative attitude and poor PPE-related practices. A high level of knowledge was associated with a positive attitude. In turn, a positive attitude was associated with good PPE practices. Welders need to be sensitized on the health risks that may arise from the non-use of PPE.

## Background

Globally, about 374 million workers are involved in non-fatal occupational accidents while 160 million people suffer from non-fatal work-related diseases (ILO, 2021). Available data indicate that more than 2.78 million workers die from work-related injuries or diseases, among which 350,000 are attributed to fatal injuries and 2 million to occupational diseases (ILO, 2021). While occupational accidents and disease rates are slowly declining in most industrialized countries, these rates are on a steady increase in developing countries. Available statistics indicate that there are about 21 fatalities per 100,000 workers and 16,000 accidents per 100,000 workers in Sub-Saharan Africa. This translates into 54,000 deaths and 42 million work-related accidents (ILO, 2015; Itiakorit, Zziwa, & Osuret, 2021).

Occupational health entails the promotion and maintenance of the highest degree of physical, mental and social well-being of workers in all occupations (ILO, 2005). Failure to promote Occupational Safety and Health (OSH) is associated with a 2.3% loss of quality life years, premature death, pain and suffering, and deterioration in mental health (ILO, 2015; WHO, 2009). Estimates by the International Labour Organisation (ILO) indicate that lost working time, worker’s compensation, interruption of production, and medical expenses cost 4% of the global Gross Domestic Product (roughly 2.8 trillion US dollars) (ILO, 2015). Despite all these negative outcomes, there is limited evidence of the burden of the problem among the more than 3 million workers whose primary occupation is welding (Husgafvel-Pursiainen & Siemiatycki, 2009; Itiakorit et al., 2021; Sabitu, Iliyasu, & Dauda, 2009b).

The ILO Constitution advocates for the protection of workers from sickness, disease and any kind of injury that is likely to arise from their employment (ILO, 2005). The Uganda Occupational Safety and Health Act 2006 also makes it mandatory for the employer to ensure the health, safety and welfare of their employees (Government of Uganda, 2006). However, compliance with OSH requirements has been reported to be low in small-scale industries (Itiakorit et al., 2021). In Uganda, small-scale industries are defined as those employing between 5 and 49 people and having total assets between UGX10 million (≈2,841 USD) but not exceeding 100 million (≈28,406 USD) (UIA, 2022). Small-scale industries employ a considerable proportion of the Ugandan population. Such industries engage in welding, spray painting, woodwork and metalwork, where protective gears are hardly used, and exposure to work-related health hazards is high (UIA, 2022; URN, 2008).

Welders in the small-scale metal workshops in Uganda engage in high-risk activities such as heavy lifting, cutting, polishing and joining metal parts, which predispose them to physical, chemical and ergonomic hazards (Atukunda et al., 2019; Itiakorit et al., 2021). The commonest technologies used in these workshops are electric arc and oxy-fuel gas welding which produce welding fumes that are detrimental to health (Joseph et al., 2017). Besides the fumes, welders in small-scale metal workshops are exposed to noise, dust, intense light, heat, electric shock, fires, vibrations from machinery, repetitive work and heavy lifting, whose risk can be exacerbated by the non-usage of personal protective equipment (PPE) and lack of administrative controls (Alexander et al., 2016; Atukunda et al., 2019; Oluwole et al., 2018). Despite this risk, there is limited evidence of their knowledge, attitude and practices toward the use of PPE. Existing studies in Uganda have particularly focused on determining the prevalence of occupational injuries and disorders and associated factors (Atukunda et al., 2019; Itiakorit et al., 2021), with limited attention on the knowledge, attitude and practices (KAP) related to PPE use.

Whereas the use of PPE is considered the last line of defence in the hierarchy of hazard control (Park, 2020), it has been identified as an important hazard control strategy in work environments where no other approach is feasible, or when the degree of safety achieved by other options is considered inadequate (Alli, 2008; Emmanuel, 2013), as was the case in small-scale metal workshops in Nansana Municipality. This study used the KAP model to assess the welder’s knowledge, attitude and practices towards the use of PPE to inform relevant strategies and interventions aimed at improving the utilization of safety measures in small-scale metal workshops. The KAP survey is a representative study of a specific population that aims to collect data on what is known, believed and done about a particular topic (Launiala, 2009; WHO, 2008). It reveals misconceptions or misunderstandings and potential barriers to desired behaviours (Launiala, 2009; WHO, 2008).

## Materials and methods

### Study area and design

A mixed-methods cross-sectional study was conducted in 50 metal workshops in Nansana Municipality in January 2019. Nansana municipality is one of the major urban local governments that was created in July 2017. It is located approximately 9.6 km from Kampala, Uganda’s capital city. The coordinates of Nansana municipality are 0°21'50.0“N, 32°31'43.0”E (Latitude: 0.363889; Longitude: 32.528611). Nansana municipality has a total population of 365,124 people; among whom 47.1% are males (UBOS, 2017). About 70.9% of persons aged 18 years and above in the municipality were engaged in some form of employment (UBOS, 2017). The municipality has a vast number of small-scale welding workshops. These employ about 3–5 employees and support the livelihoods of people in the municipality. Regarding access to healthcare services, 98.6% of the population are in less than 5 km reach of either a public or private healthcare facility while 89.0% are within a 5 km reach of a public healthcare facility.

### Study population and eligibility

This study was conducted among welders in small-scale metal workshops in Nansana municipality. In order to participate, a welder had to have worked in a small-scale metal workshop within the municipality for at least 6 months. Only welders aged 18 years and above were interviewed. We excluded welders who were too sick to participate at the time of the survey.

### Sample size and sampling procedures

The sample size for the quantitative component was determined using the Kish Leslie formula for cross-sectional studies (Kish, 1965). A z-score of 1.96 (95% CI), an error rate of 5%, and an estimated proportion of regular use of PPE of 69% (Okuga, Mayega, & Bazeyo, 2012) were used. This yielded a minimum sample size of 329 respondents. Nansana and Nabweru divisions were purposively selected due to their high number of small-scale metal workshops. A total of 25 metal workshops was selected randomly from each division. Prior to the selection of small-scale workshops, a list was first generated, after which the Ms Excel randomiser was applied for the random selection. In addition, 10 key informants (KIs) were selected purposively, based on their job positions (i.e. either managers of the health department in Wakiso district or workshop manager); level of influence and involvement in OSH; expert knowledge and experience; and willingness to share experiences.

### Data collection tools

Quantitative data were collected using structured questionnaires and observation checklists. The structured questionnaire was developed and validated by a team of occupational health experts based at the College of Health Sciences Makerere University. The questionnaire was used to obtain detailed information on socio-demographics, knowledge, attitude and practices towards the use of PPE. The observation checklist was used to obtain information on the different types of PPE, and whether the PPE were appropriate/conventional. Conversely, qualitative data were collected through face-to-face interviews with the KIs using an interview guide. The KIs interview guide consisted of open-ended questions related to broad subject areas such as availability of PPE, training on health and safety and enforcement of OSH regulations. Interviews were conducted in either Luganda (the local language) or English at the KIs’ places of work or any other place where they felt comfortable. The development of the KI interview guide was informed by a review of literature on OSH of welders (Chauhan, Anand, Kishore, Danielsen, & Ingle, 2014; Isah & Okojie, 2006; Itiakorit et al., 2021; Kumar, Dharanipriya, & Kar, 2013; Oluwole et al., 2018; Sabitu, Iliyasu, & Dauda, 2009a).

### Variable measurement

The outcome variables in the current study included the level of knowledge, attitude and practices related to PPE use. The level of knowledge was assessed by a set of 5 closed-ended questions. Study participants were asked questions about the different types of PPE, the purpose of PPE, who must wear PPE and, the existing regulations on PPE use. For each of the questions, a total score was computed according to the number of all correct responses. Each correct response was scored one point while wrong/negative response or no response was scored zero. The mean score for the level of knowledge of welders on PPE was taken as a cut-off point. Those who obtained a score above the mean (7.8) were considered as having a high level of knowledge. Respondents who scored below the mean value were categorized as having a low level of knowledge. This approach was also used by Tadesse (2016) to determine the level of awareness of occupational hazards and associated factors among welding employees in Ethiopia. Attitude was assessed by a set of 5 questions. These included attitude towards; the cost of PPE, PPE training sessions, the necessity of PPE and the possibility of being bothered by PPE, and priority when employed in a workshop. For each of the questions, a total score was computed according to the number of all correct responses. Each correct response was scored one point while wrong/negative response or no response was scored zero. Attitude was categorized as either positive or negative attitude. A mean attitude score of 1.5 was taken as a cut-off point. Respondents who obtained a score above the mean were considered as having a positive attitude. Those who scored below the mean value were considered to have a negative attitude.

A total of 4 questions were used to assess PPE-related practices. These questions elicited information on; ownership of PPE, use of PPE, undertaking PPE-related training and receiving PPE-related instructions prior to work. For each of the questions, a total score was computed according to the number of all correct responses. Each correct response was scored one point while wrong/negative response and no response were scored zero. The mean score for the practices of welders related to PPE was taken as a cut-off point and those who obtained a score above the mean score (2.3) were considered as having good practices and those below the mean were considered as having bad practices.

### Quality assurance and control measures

Research assistants were trained on the research protocol, including ethical considerations and data quality. All study tools were translated to and administered in the local language (Luganda). Prior to the main survey, data collection tools were pre-tested among 10 welders in small-scale metal workshops in Entebbe municipality. Entebbe municipality was selected as a pretest site because it shares similar characteristics with Nansana municipality. The pretest was aimed at enabling the research assistants to familiarise themselves with the data collection tools and also correct any errors that would arise. During fieldwork, data collected on each day were checked by the principal investigator for any inconsistencies to further ensure quality.

### Data management and analysis

Quantitative data collection and entry were done using a mobile data collection software, Epicollect5 and then transferred to STATA 13 for data cleaning and statistical analysis. Univariate analysis was done to determine means and frequencies. Bivariate analysis was done to determine associations between the predictor and outcome variables i.e. knowledge, attitude and practices associated with the use of PPE. A ‘modified Poisson’ regression analysis was used to assess the strength of the association between the independent/ predictor and outcome variables. At the multivariable level, variables with a *p*-value less than 0.2 were run with ‘modified’ Poisson model to generate the adjusted prevalence ratios (Zou, 2004). A *p*-value less than 0.05 was considered statistically significant. All results are summarized in tables and graphs. Qualitative interviews were conducted in either Luganda (local language) or English. Interviews were recorded to reduce recall bias by the research team. The audio files were then transcribed verbatim. Qualitative data were analyzed using NVivo 12 software.

### Ethical considerations

Ethical clearance was obtained from the Makerere University School of Public Health, Higher Degrees Research and Ethics Committee. Administrative clearance was also sought from Wakiso District local government. In addition, informed written consent was obtained from all the study participants. A consent form entailing the rationale and benefits of the study, and the rights of the participants was prepared and signed by all the study participants. Only those willing to participate in the study were interviewed. All information provided by the participants was kept confidential.

## Results

### Socio-demographic characteristics of the study respondents

A total of 329 males were interviewed. The mean age of the respondents was 27.5 ± 7.9 years. More than half, 55.6% (183/329) of the respondents were between 18 and 26 years of age, 55% (181/329) were single; 62.3% (205/329) had attained at least a secondary level of education, and welding was the main occupation for over 86% (283/329). The majority, 84.2% (277/329) of the welders were trained by apprenticeship (Table 1).

**Table 1.** Socio-demographic characteristics of the welders in small-scale industries in Nansana municipality, Uganda.

Variable	Attribute	Frequency (*n* = 329)	Percentage (%)
Age (mean = 27.5, SD = ±7.9) (years)	18–26	183	55.6
	27–35	93	28.3
	36–44	35	10.6
	Above 44	18	5.5
Marital status	Single	181	55.0
	Married	136	41.3
	Divorced	12	3.7
Education	No formal education	23	7.0
	Primary	71	21.6
	Secondary	205	62.3
	Tertiary	30	9.1
Duration of employment as a welder	Less than one year	46	14.0
	Between 1 and 5 years	167	50.8
	More than 5 years	116	35.3
Welding main occupation	Yes	283	86.0
	No	46	14.0
Mode of training for welding	Apprenticeship	277	84.2
	Formal training	52	15.8

### Knowledge related to PPE among welders among welders

Majority of the welders; 97.3% (320/329) knew that PPE was important in reducing exposure to hazards and 99.4% (327/329) were knowledgeable about at least one type of PPE. The most commonly known PPE were goggles; 98.2% (321/327) and the least known were earmuffs/earplugs; 21.4% (70/327). About, 86.6% (285/329) of the welders correctly reported the importance of PPE use. It’s important to note that none of the welders in this study was knowledgeable about regulations related to PPE use (Table 2).

**Table 2.** Knowledge about PPE and its use among welders in small-scale industries in Nansana Municipality, Uganda.

Variable	Attribute	Frequency (*n* = 329)	Percentage (%)
PPE can reduce exposure to hazards	Yes	320	97.3
	No	9	2.7
Aware of any PPE	Yes	327	99.4
	No	2	0.6
Commonly known PPE (*n* = 327)^a^	Goggles	321	98.2
	Overalls	313	95.7
	Safety boots	301	92.1
	Gloves	260	79.5
	Ear muffs/Earplugs	70	21.4
	Welding shield	75	22.9
	Welding helmet	109	33.3
	Respirators/facemasks	202	61.8
Reason why welders use PPE	Protection from hazards	285	86.6
	Directive of the manager	31	9.4
	I don’t know	8	2.4
	Others	5	1.5
Who to wear PPE	All workers	305	92.7
	Only managers/supervisor	1	0.3
	I don’t know	23	7.0
Know regulations related to PPE	Yes	0	0.0
	No	329	100.0

^a^ Multiple response questions.

Most of the key informants asserted that the welders are knowledgeable about the importance of PPE and the outcomes of non-utilization of the protective equipment. In regards to this, managers were quoted saying;

These welders know the negative outcomes of non-utilization of PPE, therefore, as an employer, there’s nothing I do to ensure that they are knowledgeable about it since they already know what to do. (Workshop manager in Nabweru division)

Yes, my welders know the injuries associated with this job and the personal protective equipment to use to prevent them. At least they know what to do in the event of an injury, although, in most cases, I put in place measures to protect them and prevent injuries. (Workshop manager in Nabweru division)

### Sensitization on occupational safety and health

Regarding sensitization and training of welders, the key informants highlighted that sensitization of welders had never been done by concerned officials at Nansana municipal council and that training has also not been effectively done in workshops.

As a municipality, we have not prioritized these small-scale welders. We have neither done inspections nor carried out sensitizations to raise awareness. The only time we cross paths with them is when we are removing their materials or displays that are trespassing into the roads. We have never been with them just for health education. (Health official, Nansana municipal council)

Managers in these small-scale workshops believe that equipping workers with the welding skill alone is enough without equipping them with knowledge on safety precautions. They usually don’t go an extra mile to tell them why they should use PPE which brings about the poor practices and negative attitude. (Health official, Nansana municipality)

### Attitude of welders toward the use of PPE among welders

About 55.6% (183/329) disagreed that PPE use should be compulsory, and 91.2% (300/329) believed that PPE use is important in reducing hazards. About 51% (161/329) of welders reported that PPE bothers them while working. Majority of the welders, 79.6% (262/329) considered salary as the first priority when employed in a metal workshop as opposed to safety/PPE. More than half, 67.2% (221/329) of the welders reported that they would not be willing to pay for PPE training sessions (Table 3).

**Table 3.** Attitude toward PPE use among welders in small-scale industries in Nansana Municipality, Uganda.

Variable	Attribute	Frequency (*n* = 329)	Percentage (%)
PPE use should be compulsory	Yes	146	44.4
	No	183	55.6
PPE is costly for nothing	Agree	127	38.6
	Disagree	191	58.1
	Neither agree nor disagree	11	3.3
Priority when employed in a workshop	Safety/PPE	65	19.8
	Salary	262	79.6
	Respect for customers	2	0.6
Attend a free PPE training session	Yes	227	69.0
	No	102	31.0
Pay for a training session on PPE use	Yes	93	28.3
	No	236	71.7
PPE bothers me when am working	Yes	143	43.5
	No	186	56.5

Findings from the key informants affirmed that some welders had a negative attitude towards training opportunities in the municipality.

Now, welding is a gold mine for the youth development programs. So, before such a group is given funds, it is a prerequisite that they are provided with information about the dangers associated with their profession. However, the biggest challenge is that these welders have a negative attitude and believe that providing them with this information is a waste of time. (Health official, Nansana municipality)

### Practices of welders related to the use of PPE among welders

About 11.9% (39/329) of the welders did not own any PPE and only 5.5% (16/329) used PPE provided by their employers. More than three-quarters, 87.2% (287/329) used PPE. Of the 287 that used PPE, 43% (126/287) did not use PPE always. The most commonly used PPEs were goggles 97.6% (280/287) and overalls 50.9% (146/287) while the least used were welding shields 0.7% (2/287) and earmuffs (0%). The reasons for non-use of PPE were; feeling uncomfortable while using PPE 57.4% (24/42), PPE being expensive 31.0% (13/42), not being required to use PPE, 50.0% (21/42), wrong size 28.6% (12/42) and forgetfulness 14.3% (6/42) (Table 4). Findings from the observation checklist revealed that 99.3% (278/280) used unconventional goggles, the majority, 85.8% (6/7) used unconventional respirators and about 95.6% (108/113) used inappropriate safety boots. All the respondents used unconventional overalls and gloves.

**Table 4.** Practices of welders related to the use of PPE among welders in small-scale industries in Nansana Municipality, Uganda.

Variable	Attributes	Frequency (*n* = 329)	Percentage (%)
Own PPE	Yes	290	88.2
	No	39	11.9
PPE Provider	Employer	16	5.52
	Self	274	94.5
Use PPE	Yes	287	87.2
	No	42	12.8
If yes, how often (*n* = 287)	Always	161	56.1
	Sometimes	126	43.9
Most commonly used PPE^a^ (*n* = 287)	Goggles	280	97.6
	Overalls	146	50.9
	Boots	113	39.4
	Gloves	22	7.7
	Ear muffs/Ear plugs	0	0.0
	Welding shield	2	0.7
	Welding helmet	3	1.1
	Respirators	7	2.4
Undertaken PPE-related training	Yes	60	18.2
	No	269	81.8
Receive PPE-related instructions prior to work	Yes	104	31.6
	No	225	68.4
Receive supervision prior to work	Yes	66	20.1
	No	263	79.9

^a^ Multiple response questions.

Most of the key informants affirmed that discomfort, wrong size and belief that a certain welding activity does not require PPE are reasons for the non-utilization of certain types of PPE.

Providing the PPE to my employees would not be very challenging but the issue is, since majority are youths, they don’t want to wear the PPE, some cut and resize the overalls to make them more fashionable. Some make it a point to quickly wear out the PPE specifically the overalls so that they can get back to wearing their clothes as opposed to the overalls. (Workshop manager, Nansana division)

Some welders tend to drop the goggles. Some weld without eye protection probably because they believe that the welding activity being performed at that time is minor. (Workshop manager, Nabweru division)

### Level of knowledge, attitude and practices toward PPE use among welders

About 61.4% (202/329) of the welders had a high level of knowledge, less than a third, 31.3% (103/329) had a positive attitude and only 37.1% (122/329) exhibited good PPE-related practices (Table 5).

**Table 5.** Level of knowledge, attitude and practices toward PPE use among welders in Nansana municipality, Uganda.

Variable	Category	Frequency (*n* = 329)	Percentage (%)
Knowledge (Mean = 7.8, SD = ±1.9)	Low level	127	38.6
	High level	202	61.4
Attitude (Mean = 1.5, SD = ±1.06)	Negative	226	68.7
	Positive	103	31.3
Practices (Mean = 2.3, SD = ±0.95)	Poor	207	62.9
	Good	122	37.1

### Factors associated with level of knowledge on PPE use among welders

Welders’ level of knowledge on PPE use was significantly associated with the welding mode of training. Welders that were trained formally (aPR = 1.52, C.I = 1.29–1.80, *p* < 0.001) were 1.5 times as likely to be knowledgeable on the use of PPE as those that were trained by apprenticeship after adjusting for other factors (Table 6).

**Table 6.** Factors associated with the level of knowledge on PPE use among welders in Nansana municipality, Uganda.

Variable	Level of knowledge				CPR at 95% CI	APRR at 95% CI	*p*-Values
	High (*n* = 202)		Low (*n* = 127)				
	*N*	%	*F*	%			
*Age*							
18–26	95	47.0	88	69.3			
27–35	67	33.2	26	20.5	1.39 (1.15–1.68)	1.21 (0.96–1.52)	0.106
36–44	26	12.9	9	7.1	1.43 (1.13–1.82)	1.05 (0.77–1.42)	0.756
Above 44	14	6.9	4	3.2	1.50 (1.13–1.99)	1.18 (0.83–1.68)	0.347
*Education level*							
Primary	35	17.3	36	28.4			
Secondary	126	62.4	79	62.2	1.24 (0.96–1.62)	1.08 (0.84–1.39)	0.558
Tertiary	26	12.9	4	3.2	1.75 (1.34–2.31)	1.08 (0.79–1.48)	0.638
No formal education	15	7.4	8	6.3	1.32 (0.90–1.94)	1.14 (0.79–1.63)	0.492
*Marital status*							
Single	90	44.6	91	71.7			
Married	104	51.5	32	25.2	1.54 (1.29–1.83)	1.22 (0.99–1.51)	0.066
Divorced	8	4.0	4	3.2	1.34 (0.88–2.05)	1.10 (0.71–1.71)	0.658
*Duration of work experience*							
Less than one year	16	7.9	30	23.6			
Between 1 and 5 years	100	49.5	67	52.8	1.72 (1.14–2.61)	1.50(0.98–2.28)	0.059
More than 5 years	86	42.6	30	23.6	2.13 (1.41–3.21)	1.52 (0.97–2.38)	0.069
*Training modes on welding*							
Apprenticeship	153	75.7	124	97.6			
Lectures/formal training modes	49	24.3	3	2.4	1.71 (1.50–1.93)	1.52 (1.29–1.80)	<0.001^a^

^a^ Considering a 95% CI, a *p*-value ≤ 0.05 was considered to be statistically significant.

PR = Prevalence Risk Ratio, aPR = Adjusted Prevalence Risk Ratio.

Findings from the qualitative interviews asserted that the level of knowledge among the welders is greatly affected by their mode of training. With regard to this, one of the municipal health officers was quoted saying;

The level of knowledge of these small-scale welders is low because many of them have learnt on the job. If you’ve not gone through the background of certain things that you do, you may not understand the importance of PPE. (Municipal health official)

### Factors associated with attitude towards PPE use among welders

After adjusting for confounders, the attitude toward PPE use was significantly associated with the following variables; level of education, duration of work as a welder, the welders and level of knowledge (high vs low). Welders who had tertiary education (aPR = 2.31, C.I = 1.28–4.14, *p*-value = 0.005) were 2.3 times as likely to have a positive attitude towards the use of PPE as those with a primary level of education. Welders with a working experience of between one year and 5 years (aPR = 2.37, C.I = 1.01–5.55, *p*-value = 0.047) and above 5 years (aPR = 2.79, C.I = 1.13–6.89, *p*-value = 0.027) were 2.4 times and 2.8 times as likely to have a positive attitude as those who had a working experience of less than one year respectively. Welders with a high level of knowledge (aPR = 1.73, C.I = 1.13–2.65, *p*-value = 0.011) were 1.7 times as likely to have a positive attitude toward the use of PPE as those with a low level of knowledge (Table 7).

**Table 7.** Factors associated with attitude toward PPE use among welders in Nansana municipality, Uganda.

Variable	Attitude				PR (95% CI)	aPR (95% CI)	*p*-Values
	Positive (103)		Negative (226)				
	*N*	%	*N*	%			
*Age*							
18–26	49	47.6	134	59.3			
27–35	27	26.2	66	29.2	1.08 (0.73–1.62)	0.62(0.40–0.97)	0.034
36–44	18	17.5	17	7.5	1.92 (1.28–2.87)	0.98(0.57–1.67)	0.936
Above 44	9	8.7	9	4.0	1.87 (1.11–3.14)	1.07(0.56–2.04)	0.841
*Education level*							
Primary	15	14.6	56	24.8			
Secondary	61	59.2	144	63.7	1.40 (0.86–2.32)	1.25(0.76–2.04)	0.385
Tertiary	21	20.4	9	4.0	3.31 (1.99–5.50)	2.31(1.28–4.14)	0.005^a^
No formal education	6	5.8	17	7.5	1.23 (0.54–2.81)	0.88(0.42–1.86)	0.738
*Marital status*							
Single	46	44.7	135	59.7			
Married	51	49.5	85	37.6	1.48 (1.06–2.05)	1.07 (0.73–1.57)	0.713
Divorced	6	5.8	6	2.7	2.93 (0.90–9.55)	1.41(0.70–2.83)	0.331
*Duration of work as a welder*							
Less than one year	5	4.9	41	18.1			
Between 1 and 5 years	51	49.5	116	51.3	2.81 (1.19–6.64)	2.37(1.01–5.55)	0.047^a^
More than 5 years	47	45.6	69	30.5	3.73 (1.58–8.79)	2.79(1.13–6.89)	0.027^a^
*Modes of training on welding*							
Apprenticeship	73	70.9	204	90.3			
Formal training	30	29.1	22	9.7	2.19 (2.61–2.97)	1.20 (0.82–1.75)	0.348
*Level of knowledge*							
Low	23	22.3	104	46.0			
High	80	77.7	122	54.0	2.19 (1.45–3.29)	1.73 (1.13–2.65)	0.011^a^

^a^ Considering a 95% CI, a *p*-value ≤ 0.05 was considered to be statistically significant.

PR = Crude Prevalence Ratio, aPR = Adjusted Prevalence Ratio.

### Factors associated with practices related to the use of PPE among welders

Multivariate analysis revealed that the practices related to PPE use were significantly associated with welding mode of training and attitude. Welders that were trained formally (lectures) (aPR = 2.91, C.I = 2.14–3.96, *p* < 0.001) were 2.9 times as likely to have good practices as those that were trained by apprenticeship. Welders with a positive attitude (aPR = 1.45, C.I = 1.08–1.94, *p*-value = 0.013) were 1.45 times as likely to have good practices as welders with a negative attitude (Table 8).

**Table 8.** Factors associated with practices related to PPE use among welders in Nansana Municipality, Uganda.

Variable	Practice score				PR at 95% CI	aPR (95% CI)	*p*-Values
	Good (122)		Poor (207)				
	*N*	%	*N*	%			
*Age*							
18–26	66	54.1	117	56.5			
27–35	31	25.4	62	30.0	0.92 (0.65–1.30)	0.97 (0.69–1.36)	0.860
36–44	17	13.9	18	8.7	1.35 (0.92–4.89)	0.95 (0.64–1.42)	0.806
Above 44	8	6.6	10	4.8	1.23 (0.71–2.14)	1.02 (0.61–1.70)	0.926
*Education level*							
Primary	17	13.9	54	26.1			
Secondary	76	62.3	129	62.3	1.87 (1.01–3.46)	1.27 (0.80–2.02)	0.308
Tertiary	22	18.0	8	3.9	3.06 (1.92–4.89)	1.31 (0.76–2.27)	0.330
No formal education	7	5.7	16	7.7	1.27 (0.60–2.68)	1.25 (0.58–2.70)	0.570
*Marital status*							
Single	61	50.0	120	57.9			
Married	56	45.9	80	38.7	1.22 (0.92–1.63)		
Divorced	5	4.1	7	3.4	1.24 (0.61–2.49)		
*Duration of work as a welder*							
Less than one year	14	11.5	32	15.5			
Between 1 and 5 years	60	49.2	107	51.7	1.18 (0.73–1.91)		
More than 5 years	48	39.3	68	32.9	1.36 (0.83–2.22)		
*Training modes*							
Apprenticeship	76	62.3	201	97.1			
Lectures/formal training modes	46	37.7	6	2.9	3.22 (2.60–4.00)	2.91 (2.14–3.96)	< 0.001^a^
*Level of knowledge*							
Low	38	31.2	89	43.0			
High	84	68.9	118	57.0	1.39 (1.02–1.90)	0.87 (0.60–1.26)	0.464
*Attitude*							
Negative	66	54.1	160	77.3			
Positive	56	45.9	47	22.7	1.86 (1.42–2.43)	1.45 (1.08–1.93)	0.013^a^

^a^ Considering a 95% CI, a *p*-value ≤ 0.05 was considered to be statistically significant.

PR = Prevalence Ratio, aPR = Adjusted Prevalence Ratio.

## Discussion

This study established the level of knowledge, attitude and practices related to PPE use among welders in Uganda. It highlights gaps in the occupational health and safety of welders in Nansana Municipality, Wakiso district. The study revealed that a vast majority of the respondents were trained by apprenticeship. Joining a formal vocational institution to undertake the welding and fabrication course requires academic credentials which many welders may not have had at the start of their occupation. Besides, vocational institutes charge fees which may not be affordable, in addition to their limited distribution. A review of literature indicates contrasting findings regarding the training of welders in low-income countries. Although our findings are consistent with those reported by Joseph et al. (2017) among welders in the informal sector in India, which indicated that the majority were trained by apprenticeship, this is in contrast to the evidence generated by Alexander et al. (2016), which indicated that almost all welders in India had neither any formal training nor apprenticeship.

The current study revealed that a considerable proportion of welders had a high level of knowledge of PPE. This could be because all the welders in this study had at least received a certain form of training (either formal or informal) on PPE. Also, welders who were formally trained had a higher level of knowledge and better PPE-related practices compared to those trained by apprenticeship. Formal training institutions incorporate OSH into their curricula which equips workers with knowledge of safety measures and occupational hazards. The high level of knowledge on PPE reported in the current study is consistent with that reported among welders in Ethiopia (Beyene, Tetemke, & Yetum, 2019), while the relationship between the nature of training and awareness of occupation hazards has been reported in Nigeria (Sabitu et al., 2009b). Findings generated by our study indicate the need to promote formal training as a measure of improving knowledge, attitude and PPE-related practices among welders.

Generally, welders were found to be knowledgeable about the different types of PPE. The most commonly known PPE were goggles and the least known were earmuffs/earplugs. This could be attributed to the higher likelihood of occurrence and severity of eye-related injuries compared to hearing impairment, thus necessitating the need for goggles for protection. Similar findings have been reported among welders in Nigeria (Gwomson et al., 2018). It’s worthy reporting that none of the welders in this study was knowledgeable about regulations related to PPE use. This could be because of the limited training opportunities and the complete absence of sensitization on OSH policies and regulations for the welders by the relevant authorities. This implies that welders are not aware of their rights including the right to demand for a safe working environment, and the employers’ duty to provide adequate and suitable PPE as stipulated in sections 13 (g) and 35 of the Uganda Occupational Safety and Health Act (Government of Uganda, 2006).

Most welders in the current study had a negative attitude towards PPE use. This could be attributed to the lack of regular training on PPE use and support supervision in small-scale metal workshops. The negative attitude could translate into poor practices such as non-utilization and the use of inappropriate PPE that may put the welders at risk of injuries. Particularly, the majority considered salary as the first priority when employed in a metal workshop as opposed to safety/PPE. Our findings imply that a significant proportion of the welders in our study had a low-risk perception of occupational hazards and, did not understand the need for use of PPE. More than two-thirds of the respondents were not willing to pay for a training session on the use of PPE, and only 69% expressed willingness to attend free training. This could be because the majority of the welders believed they had adequate knowledge of PPE due to their working experience. This attitude implies a lack of willingness for the advancement of knowledge of OSH by some welders, therefore continuous health education is needed to change this perception.

Attainment of tertiary education was associated with a positive attitude towards PPE use. A higher level of education fosters a better understanding of the safety measures and thus promotes a positive attitude (Nasab, Tavakoli, Ghofranipour, Kazemnejad, & Khavanin, 2009). Similarly, an increase in the years of experience was associated with a positive attitude towards PPE use. An increase in years of experience implies more training opportunities and increased exposure to occupational hazards, which ultimately improves the level of knowledge and attitude towards PPE use. Similar findings have been reported in earlier studies (ILO, 2015; Itiakorit et al., 2021). Welders with a high level of knowledge were more likely to have a positive attitude compared to those with a low level of knowledge. An increase in the level of knowledge leads to a better understanding of the inherent risks related to occupation and translates into a positive attitude towards the use of PPE (Mary, Anyalewechi, Chukwudi, Christian, & Maryjane, 2020).

Majority of the welders owned PPE. PPE ownership is important because it ensures availability when needed for use by the welders. However, almost all welders bought their own PPE because it was a prerequisite for being hired. This implies that employers don’t prioritize PPE provision and yet, the OSH act of Uganda states that it is the responsibility of the employer to provide free protective equipment to their workers (Government of Uganda, 2006). Almost half of the welders did not use PPE regularly. This could be attributed to inadequate enforcement of OSH regulations in the small-scale metal workshops which affects compliance with safety measures. Irregular use of PPE implies that welders are exposed to occupational hazards. The most commonly used PPEs were goggles while the least used were earmuffs. This correlates with the fact that goggles were reported to be the most known compared to other PPEs. It could also be because goggles are the most affordable and are perceived as the most important PPE for welding due to the high likelihood and severity of eye-related injuries. This however implies that welders in this study are at risk of injuries due to inhalation of welding smoke, cuts, burns and noise since the majority did not utilize respirators, gloves, boots, and earmuffs. This is consistent with findings from Nigeria which indicated that goggles (98%) were the most used PPE in comparison to ear muffs (12.9%) (Yetunde et al., 2018).

In this study, the use of inappropriate PPE was high among welders. This could be because the non-conventional PPE are more affordable, available and comfortable compared to the conventional types. Thus almost all of the welders in this study used PPEs that offer minimal or no protection at all from the hazards and were at risk of injuries. These findings are quite similar to those in previous studies done in developing countries where the majority of the welders did not use the appropriate recommended PPE (Alexander et al., 2016; Budhathoki, Singh, Sagtani, Niraula, & Pokharel, 2014; Hassan, Nasir, Anwar, & Talib, 2017; Zgambo, 2015). The study revealed that the majority (62.9%) of the welders had poor PPE-related practices. This could be because of the negative attitude that the majority of the welders had. It could also be attributed to the limited sensitization opportunities available for the welders in Nansana municipality, and the lack of regular training and support supervision at work. Welders with a positive attitude were more likely to exhibit good practices than those with a negative attitude. It’s therefore important to not only target the improvement of welders’ knowledge, but also their attitude towards PPE to improve practices.

## Conclusion

A significant proportion of welders had a high level of knowledge, less than a third had a positive attitude, and slightly more than a third exhibited good PPE-related practices. Knowledge of the use of PPE significantly varied by type of training. Attitude toward PPE use varied across the level of education, work experience, and level of knowledge. A positive attitude and undergoing a formal welding and fabrication course predicted better PPE-related practices. Our study reaffirms the relationship between knowledge, attitude and practices. Welders who were more knowledgeable were more likely to have a positive attitude and to exhibit better PPE-related practices. The current study underscores a dire need for the relevant stakeholders to sensitize welders on occupational risks, their OSH rights and the responsibilities of the employers in upholding OSH. Attention should be given to inexperienced welders and those trained informally (e.g. through apprenticeship). In addition, continuous training and sensitization of welders on PPE ought to be coupled with support supervision and enforcement. Coupling these interventions will not only improve knowledge and influence attitude but will also improve PPE-related practices.

## Data Availability

The datasets used during this study are available from the corresponding author upon reasonable request.
